# The expression of TRIAD1 and DISC1 after traumatic brain injury and its influence on NSCs

**DOI:** 10.1186/s13287-018-1024-9

**Published:** 2018-11-08

**Authors:** Rui Jiang, Qianqian Liu, Hui Zhu, Yong Dai, Junzhong Yao, Yazhou Liu, Pei Pei Gong, Wei Shi

**Affiliations:** 1 Department of Neurosurgery, Affiliated Hospital of Nantong University, Nantong, 226001 China; 2 Jiangsu Clinical Medicine Centre of Tissue Engineering and Nerve Injury Repair, Affiliated Hospital of Nantong University, Nantong, 226001 China

**Keywords:** TRIAD1, DISC1, Traumatic brain injury, NSCs, Proliferation, Differentiation

## Abstract

**Background:**

After cerebral injury, the proliferation and differentiation of neural stem cells are important for neural regeneration.

**Methods:**

We used the SD rat to establish the traumatic brain injury model. Then, we verified molecular expression, interaction through Western blot, immunoprecipitation (IP), immunofluorescence, and other methods. All data were analyzed with Stata 8.0 statistical software.

**Results:**

We showed for the first time that the interaction of TRIAD1 and DISC1 plays an important role in neural stem cell proliferation and differentiation after traumatic brain injury. In a rat model of traumatic brain injury, we found that the expression of TRIAD1 increased progressively, reached a peak at 3 to 5 days, and then decreased gradually. While the expression level of DISC1 was correlated with TRIAD1, its expression level at 3 days was significantly lower than at other time points. Immunofluorescence on frozen brain sections showed that TRIAD1 and DISC1 are co-localized in neural stem cells. Immunoprecipitation data suggested that TRIAD1 may interact with DISC1. We transfected 293T tool cells with plasmids containing truncated fragments of TRIAD1 and DISC1 and used additional IPs to reveal that these two proteins interact via specific fragments. Finally, we found that overexpressing TRIAD1 and DISC1 in primary neural stem cells, via lentiviral transfection, significantly affected the proliferation and differentiation of those neural stem cells.

**Conclusions:**

Taken together, these data show that the expression of TRIAD1 and DISC1 change after traumatic brain injury and that their interaction may affect the proliferation and differentiation of neural stem cells. Our research may provide a sufficient experimental basis for finding molecular targets for neural stem cell proliferation and differentiation.

**Trial registration:**

We did not report the results of a health care intervention on human participants.

## Background

Traumatic brain injury (TBI) causes serious adverse outcomes including death and disability in developed and developing countries [1, 2]. The pathophysiology of TBI includes primary injury caused by direct damage through the initial source of trauma and secondary injury caused by biochemical responsive cascades after subdural hemorrhage and cerebral contusion, such as hypoxia, inflammatory responses, axonal stretch injury, and apoptotic cell death [3]. It is well known that neural stem cells (NSCs) have the characteristics of clear location, simple composition, and immobilized pathways [4, 5]. In addition, NSCs can stably differentiate into astrocytes, neurons, oligodendrocytes, and other cell types [6, 7]. Therefore, cell replacement therapy via transplantation of exogenous NSCs or activation of endogenous NSCs is now a cutting-edge treatment for TBI worldwide.

It should be noted that the use of NSCs to treat brain damage still faces many difficulties. One of the most critical is the efficacy of transplanted or endogenous NSCs in nerve regeneration. Ensuring the survival and proliferation of NSCs, as well as their differentiation into neurons or oligodendrocytes [8], is among the other major problems restricting the success of this treatment option in TBI. However, studies have shown that NSCs can maintain cell viability and differentiate more quickly under physiological conditions if provided with certain external factors or genetic modifications [9].

DISC1-encoded scaffold proteins can regulate neurogenesis, as well as the proliferation, migration, and differentiation of NSCs [10]. Ju Young Kim and colleagues show that DISC1 can bind the Girdin protein and affect the proliferation of NSCs by regulating downstream signaling pathways [11]. Others also found that the DISC1 protein can regulate embryonic cortical development [12]. DISC1 itself is a transcription factor, which may be involved in the transcriptional regulation of NSC-related molecules, thereby affecting the differentiation of NSCs [13].

The ubiquitin proteasome system (UPS) plays an important role in the process of protein catabolism [14]. The UPS involves the coordinated action of E1 ubiquitin-activating enzymes, E2 ubiquitin-conjugating enzymes, and E3 ubiquitin ligases, of which each family includes a variety of members. The UPS decreases protein levels by the ubiquitination and subsequent degradation of different target proteins. It is now believed that TBI modifies many proteins in the system and influences the ubiquitination of their protein targets, which is an important way to regulate neuronal function [15–17]. It was found that the expression of ubiquitin proteases, such as Pirh2, XIAP, and SCYL1-BP1, was changed after TBI. Additionally, neuronal apoptosis and activation of astrocytes can be regulated by altering the ubiquitination level of downstream molecules, such as P27 [18–20].

TRIAD1 (ARIH2, Ariadne RBR E3 ubiquitin protein ligase 2) is an E3 ubiquitin ligase that is a member of the UPS family and is involved in many important intracellular events, such as protein stability regulation and cell cycle regulation. It has been proved that TRIAD1 plays an important role in the hematopoietic system and inhibits the proliferation of U937 cells and the formation of cell colonies [21–23]. It also affects embryogenesis through hematopoietic stem cells [24].

Here, we found for the first time that the expression of TRIAD1 and DISC1 change after TBI, and the interaction between TRIAD1 and DISC1 may affect the proliferation and differentiation of NSCs.

## Methods

### Animals and the TBI model

Male Sprague–Dawley rats (200–250 g body weight) were obtained from the Experimental Animal Center of Nantong University, Nantong, China. All the procedures were in strict accordance with the institutional guidelines of Nantong University, which complies with international rules and policies. Ethics in accordance with the ARRIVE (Animal Research: Reporting In Vivo Experiments) guidelines were followed in the animal experiments and approved by the Animal Care and Use Committee of Nantong University, Nantong, China. All the surgeries were performed under anesthesia, and all efforts were made to minimize suffering and the number of rats used in this study.

TBI was induced following a previously described model of cortical contusion trauma for the rat [25]. The animals were anesthetized with 10% chloral hydrate (400 mg/kg, i.p.). After placement of the rat in a stereotactic frame, a right parietal craniotomy (3.5 mm posterior and 2.5 mm lateral to bregma, diameter 5 mm) was made with a drill under aseptic conditions. A steel rod weighing 20 g with a flat end and diameter of 4.5 mm was dropped onto a piston resting on the dura from a height of 25 cm. The piston was allowed to compress the brain tissue to a depth of 2.5 mm and was removed immediately after the contusion. The sham-operated rats were surgically treated with right parietal craniotomies without brain injury. Rectal temperature was maintained within the range of 37 ± 0.5 °C with a heating pad. After the trauma procedure, the rats were returned to their cages and kept at room temperature.

### Western blot analysis

To prepare for Western blot analysis, frozen brain tissue was weighed and then minced with scissors on ice. Samples were mixed with lysis buffer (50 mM Tris, pH 8.0, 5 mM EDTA, 150 mM NaCl, 1% sodium deoxycholate, 1% NP-40, 0.2% Triton X-100, and 1× complete protease inhibitor cocktail (Roche Diagnostics, Basel, Switzerland)). The primary NSCs were collected from plates with phosphate-buffered saline (PBS) solution, resuspended with RIPA buffer (50 mM Tris, pH 7.4, 1% NP-40, 150 mM NaCl, 0.5% sodium deoxycholate, 0.1% sodium dodecyl sulfate (SDS), and 1× complete protease inhibitor cocktail). All these steps were performed on ice. The protein lysates were separated with 10% SDS-PAGE and transferred to a polyvinylidene difluoride (PVDF) membrane (Millipore Corporation, Billerica, USA). The membrane was blocked with 5% skim milk for 2 hours (h) at room temperature and then incubated with corresponding antibodies. The protein bands were ultimately visualized with an Odyssey infrared Western Blot Imager (LICOR, Lincoln, USA).

### Co-immunoprecipitation (Co-IP)

Cells were transfected with 2 μg of DNA with 6 μl of Fugene-6 transfection reagent (Roche Diagnostics) in 100 μl of serum-free medium and then allowed to grow for 36–48 h. The cells were lysed with lysis buffer (50 mM Tris-HCl, pH 7.5, 150 mM NaCl, 5 mM EDTA, 15 mM MgCl2, 0.1% NP-40, and Protease Inhibitor Cocktail [Roche, Mannheim, Germany]) and then clarified by centrifugation. The supernatant was pre-cleared using protein G-agarose beads (Roche) for 4 h at 4 °C and then incubated with 5 μl antibody and beads overnight at 4 °C. The immunocomplexes were pelleted and washed three times with lysis buffer. The immunocomplexes and cell lysate protein were used for Western blot analysis.

### Immunohistochemistry

The sections were blocked with confining liquid consisting of 10% donkey serum, 1% bovine serum albumin (BSA), 0.15% Tween-20, and 0.3% Triton X-100 for 2 h at room temperature. They were then incubated with TRIAD1 antibody (rabbit anti-TRIAD1, 1:100, Abcam, UK) and DISC1 antibody (mouse anti-DISC1, 1:100, Santa Cruz Biotechnology, USA) overnight at 4 °C. Next, the sections were incubated with the corresponding second antibody for 30 minutes (min) at room temperature, and then with the coloring liquid mixture (0.02% diaminobenzidine tetrahydrochloride (DAB), 0.1% PBS, and 3% H_2_O_2_). Finally, the sections were dehydrated and covered with coverslips. We examined the sections from each group at higher magnification and counted those cells with strong or moderate brown staining as positive cells, and weak or no staining as negative cells.

### Immunofluorescence

At defined survival times, rats (*n* = 3 per time point) were anesthetized and perfused with 500 ml of 0.9% saline followed by 4% paraformaldehyde. After perfusion, the brains were extracted and fixed in the same fixative for 3 h. Then, they were placed in 20% sucrose for 2–3 days, followed by 30% sucrose for another 2–3 days. Finally, the tissue was embedded in OTC compound (optimal cutting temperature compound), and frozen cross sections of 6-μm thickness were prepared. All sections were stored at − 20 °C. Slide-mounted sections were kept in an oven at 37 °C for 30 min and then rinsed twice in 0.01 M PBS for 5 min. All sections were incubated in a blocking solution containing 10% normal donkey serum (Jackson, West Grove, USA), 3% (*w*/*v*) BSA, 0.1% Triton X-100, and 0.05% Tween-20, for 2 h at room temperature to avoid non-specific staining. Then, they were exposed to the TRIAD1 antibody (rabbit anti-TRIAD1, 1:100, Abcam, UK), DISC1 antibody (mouse anti-DISC1, 1:100, Santa Cruz Biotechnology, USA), or Nestin antibody (anti-rabbit/mouse, 1:100, Abcam, UK) overnight at 4 °C. Then, sections were treated with a mixture of FITC- and TRITC-conjugated secondary antibodies for 2 h at 4 °C. To detect the morphology of cells, sections were also stained with DAPI (0.1 mg/ml in PBS; Sigma) for 40 min at 30 °C. The stained sections were examined with a Leica fluorescence microscope (Leica, DM 5000B; Leica CTR 5000; Leica, Germany).

### Isolation, purification, and culture of NSCs

Embryonic NSCs were isolated from embryonic day 16 (E16) rat cortex. Cells were maintained in floating culture in proliferation medium containing 20 ng/ml basic fibroblast growth factor (bFGF), 20 ng/ml epidermal growth factor (EGF), and 2% B27 supplement, and were passaged every 4–6 days. These NSCs can still proliferate and are able to self-renew.

Adult NSCs were isolated and cultured in the same way as the embryonic NSCs, with some modifications. In brief, the dentate gyri of 2-month-old female mice (three mice were used in each primary isolation experiment) were dissected and digested with 0.125% trypsin (Gibco) and 250 U/ml DNase I (Sigma-Aldrich) at 37 °C for 20 min, and the undifferentiated progenitors were enriched by centrifugation with Percoll. Adult NSCs were maintained in floating culture in proliferation medium similar to that of embryonic NSCs. The proliferation and self-renewal capacity, as well as multi-differentiating potential of adult NSCs, were as easily identified as in the embryonic NSCs. Adult NSCs in their second to fourth passages were used in this study.

For lentivirus transfection and cell co-culture experiments, NSCs were plated at a cell density of 1 × 10 cells/cm^2^ and were cultured as a monolayer. The co-culture model was set up with astrocytes exposed to lipopolysaccharide to simulate the process of NSC mobilization after TBI. The identified NSCs were gently pipetted into single-cell suspensions, seeded on polylysine-coated sterile coverslips (2 cm^2^) in 24-well plates, and cultured in medium containing 10% fetal bovine serum and a 1:1 ratio of DMEM and F12.

### Plasmid and transfection

The plasmid was obtained from Genechem Biotechnologies Co., Ltd. The truncated TRIAD1 (1–246; 247–493) and DISC1 (1–1008; 1009–2046) mutant fragments were created from wild-type TRIAD1 and DISC1. All of the transfection assays were performed with Lipofectamine 2000 transfection reagent (Invitrogen, Carlsbad, CA, USA) according to the manufacturer’s protocol.

### Statistical analysis

All data were analyzed with Stata 8.0 statistical software (Systat Software Inc., San Jose, CA, USA). All values are expressed as the mean ± SEM. The data were compared using Student’s *t* test and *p* < 0.05 was considered statistically significant. Each experiment consisted of at least three replicates per condition.

## Results

### Expression patterns of TRIAD1 and DISC1 after cortical injury

**Fig. 1:**
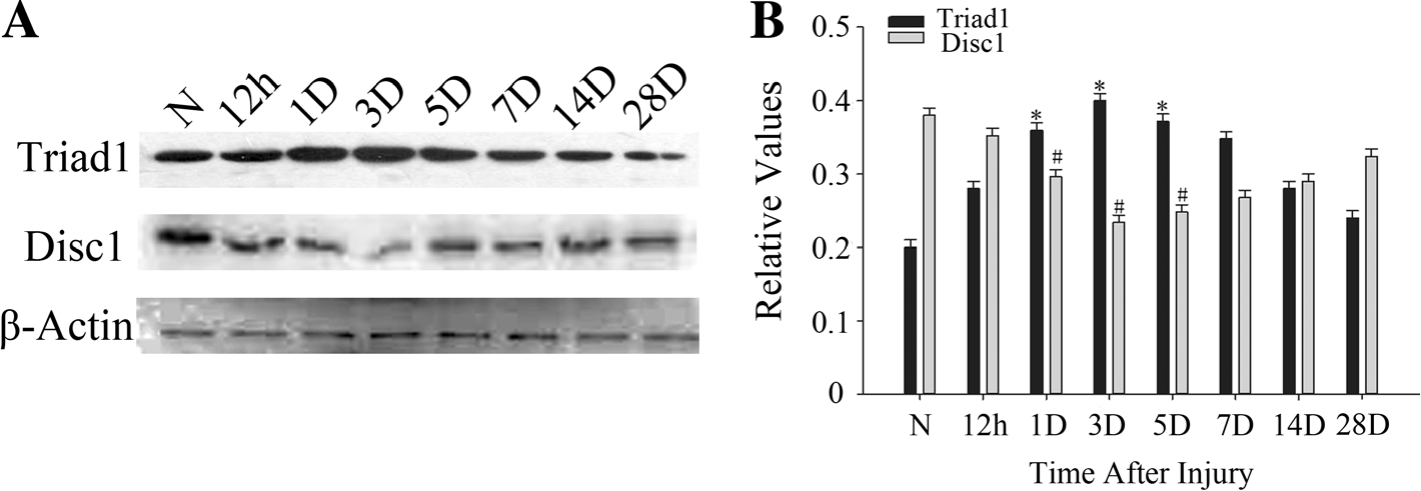
Expression patterns of TRIAD1 and DISC1 after cortical injury. The expression of TRIAD1 and DISC1 in rat cortex. **a** Western blot analysis was used to measure the expression of TRIAD1 and DISC1 at indicated time points in the rat cortex. **b** Semi-quantitative analysis of TRIAD1 and DISC1 in cortices.*,#*P* < 0.05, vs. normal group (*n* = 6)

An adult rat TBI model was used to explore the possible role of TRIAD1 and DISC1 after brain injury. We used Western blotting analysis to investigate the expression of TRIAD1 and DISC1 in the brain after injury. The initial level of TRIAD1 was relatively low throughout the brain, but it progressively increased from 12 h after TBI till its peak at day 3 (*p* < 0.05), and then gradually decreased to normal levels (Fig. 1). DISC1 expression gradually decreased starting at 12 h following TBI, with its lowest point at day 3 compared with the control group (Fig. 1a). TRIAD1 and DISC1 showed opposite expression patterns, which suggest that TRIAD1 could have a direct or indirect connection with DISC1.

**Fig. 2:**
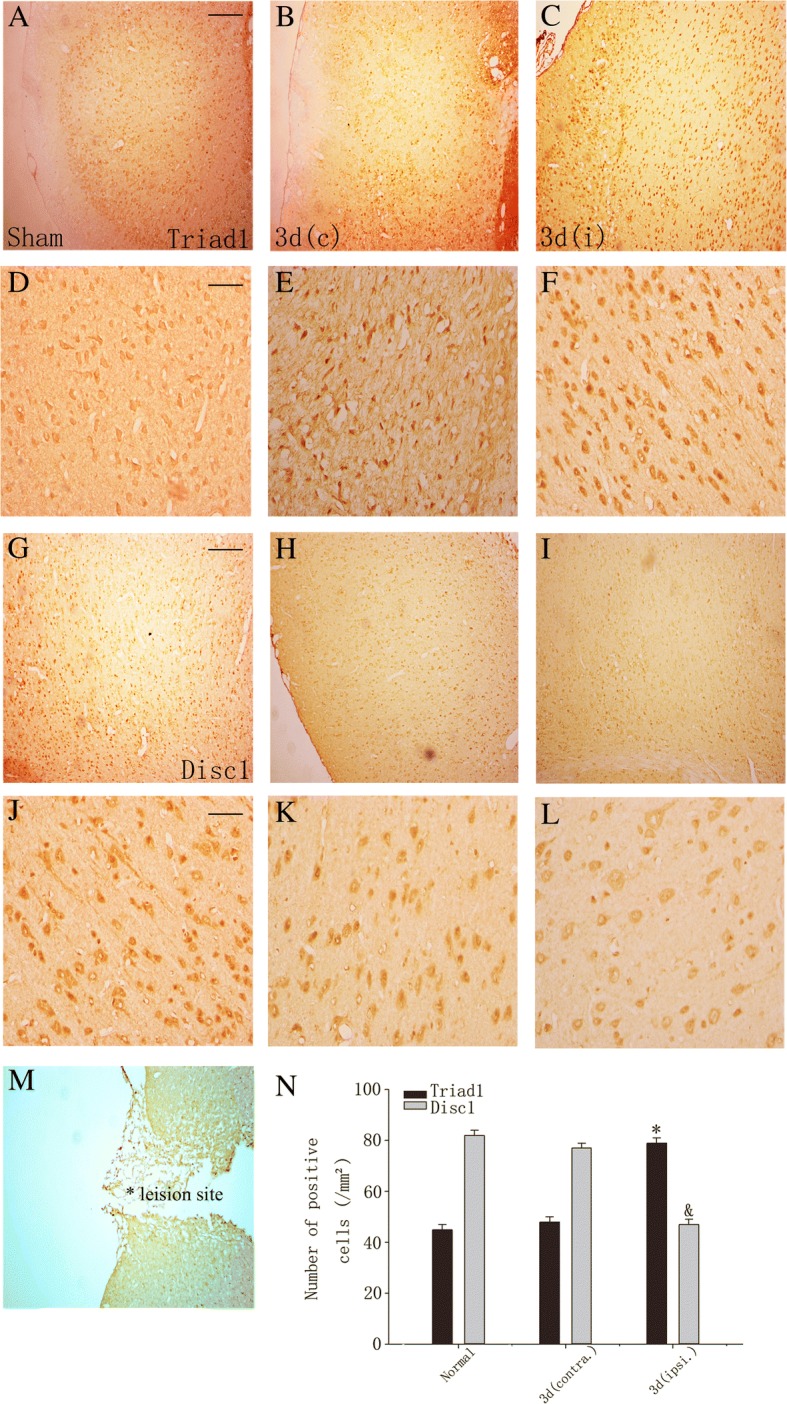
Distribution of TRIAD1- and DISC1-positive cells in rat cortex. TRIAD1 and DISC1 immunohistochemistry of rat brain cortex at 3 days after TBI. TRIAD1 immunohistochemical staining respectively in the cortex of normal rat (**a**), and contralateral (**b**) and ipsilateral (**c**) cortices of TBI rat at 3 days after injury. And DISC1 is shown in **g**–**i**. **d**–**f** and **j**–**l** Local magnification view of **a**–**c** and **g**–**i**, respectively. **m** The image of the lesion site. Scale bars, 200 μm for **a**–**c**, **g**–**i**; 50 μm for **d**–**f**, **j**–**l**. **n** Quantitative analysis of TRIAD1- and DISC1-positive cells in normal, contralateral, and ipsilateral brain cortices (*n* = 4). *,& *P* < 0.05, vs. two control groups

To investigate the distribution of TRIAD1 and DISC1 after TBI, we used immunohistochemical staining on cryosections of the brain. As shown in Fig. 2, at 3 days after TBI, the expression of TRIAD1 was markedly increased in the area surrounding the injury in the ipsilateral cortex (Fig. 2c, f) while relatively weak expression was observed in the contralateral cortex and in the sham-operated cortex (Fig. 2a, b, d, and e). DISC1 expression showed the opposite trend. The expression pattern of both proteins evaluated by cell counting was consistent with the Western blot results (Fig. 2n). These changes show an upregulation of TRIAD1 and a downregulation of DISC1 expression after TBI.

**Fig. 3:**
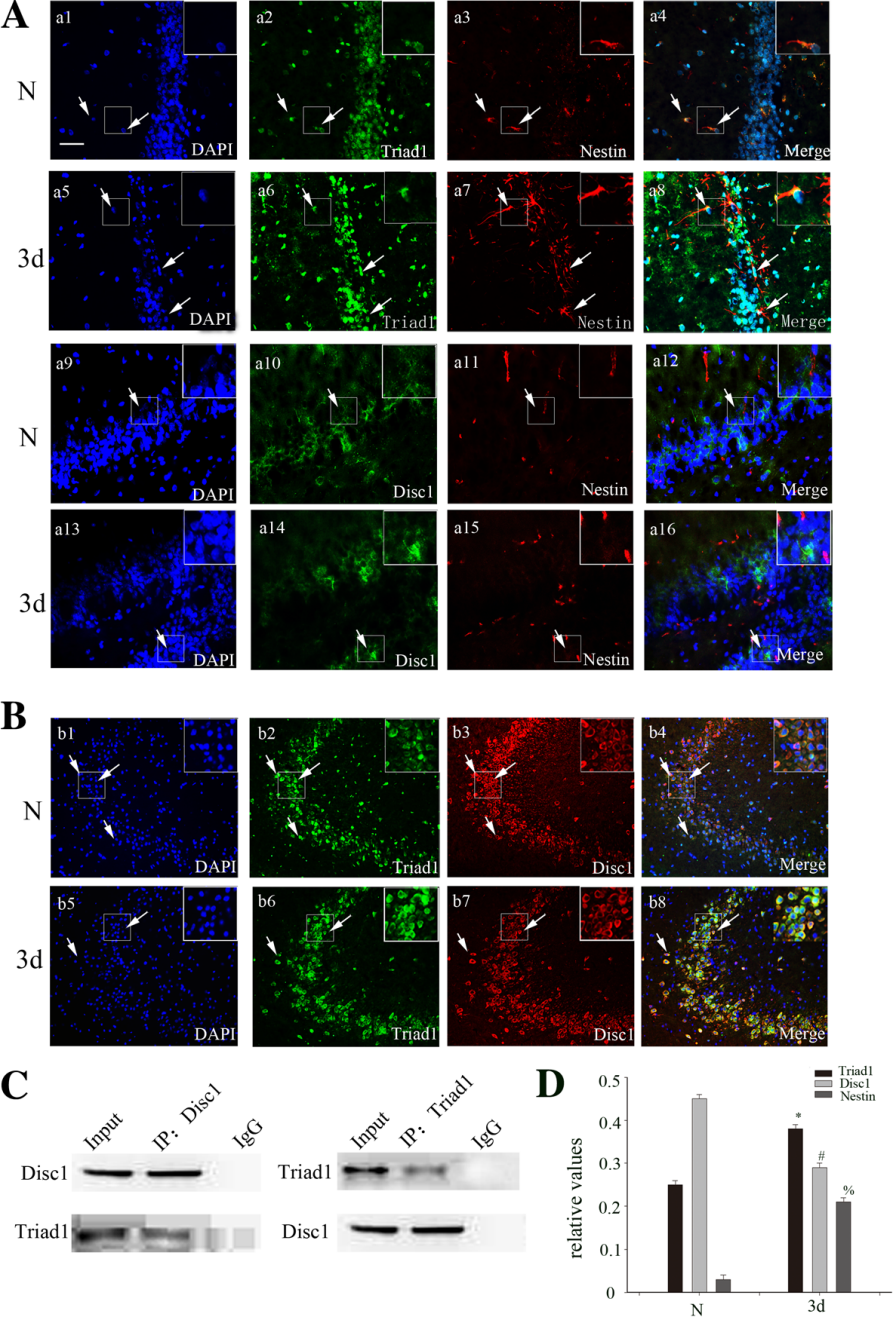
TRIAD1 co-localized with DISC1 in NSCs and could interact with each other. **a** In the adult rat brain hippocampus at 3 days after TBI, horizontal sections were labeled for Nestin (red, a3, a7) and TRIAD1 (green, a2, a6). The arrows show the co-localization of TRIAD1 and Nestin (a4, a8). Double immunofluorescence staining for Nestin (red, a11, a15) and DISC1 (green, a10, a14). The arrows show the co-localization of DISC1 and Nestin (a12, a16). **b** Double immunofluorescence staining for TRIAD1 (green), DISC1 (red), and the nuclear marker DAPI (blue). The white color visualized in merged images represents the co-localization of TRIAD1 and DISC1 (arrow). **c** Immunoprecipitation showed that the interaction of TRIAD1 and DISC1 could interact with each after TBI. **d** Statistical results for the expression of TRIAD1, DISC1, and Nestin in the hippocampus 3 days after brain injury compared with the sham group. *,#,%*P* < 0.05 indicates significant differences compared to the sham group. Scale bars, 50 μm

In order to visualize the distribution of both proteins together, we used immunofluorescence in the cerebral cortex after brain injury. Immunofluorescent labeling showed that TRIAD1 and DISC1 co-localize with each other in the dentate gyrus (DG) of the hippocampus (Fig. 3a). This suggests that TRIAD1 may interact with DISC1 in brain. Besides, we also found that TRIAD1 and DISC1 co-localized with Nestin in the DG region (Fig. 3b). Nestin is a common marker for NSCs, indicating that both TRIAD1 and DISC1 are present in this cell type after TBI.

The previous results allude to a potential interaction between TRIAD1 and DISC1. In order to further explore the relationship between them, we used co-IP to show that DISC1 interacts with TRIAD1 (Fig. 3c).

### The expression and distribution of TRIAD1 and DISC1 in primary neural stem cells co-cultured LPS-astrocytes

**Fig. 4:**
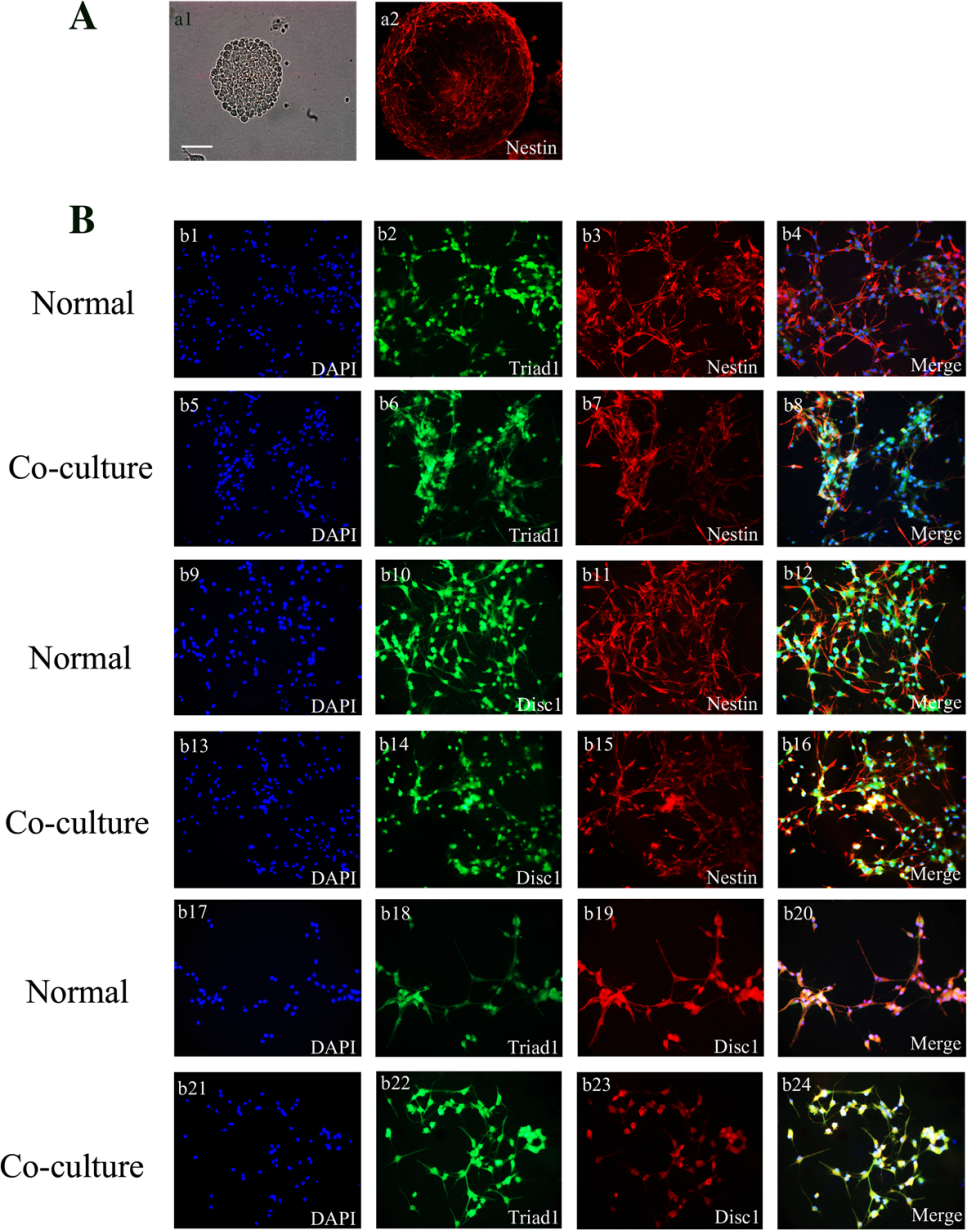
TRIAD1 co-localized with DISC1 in the primary NSCs. **a** Neurospheres were cultured as low-density cell suspensions in the presence of epidermal growth factor and basic fibroblast growth factor. And neurosphere was observed by a light microscope (a1, scale bar: 50 μm). Then, neurospheres were planted on coverslips and the expression of Nestin was detected (a2). **b** NSCs co-cultured with cm of LPS-astrocytes. Then immunofluorescence was performed to detect TRIAD1 (green, b2, b6; green, b18, b22), DISC1 (green, b10, b14; red, b19, b23), and NESTIN (red, b3, b7, b11, b15). The nuclei were stained with DAPI (blue, b1, b5, b9, b13, b17, b21). (b4) and (b8) show the co-localization of TRIAD1 (green) and NESTIN (red). (b12) and (b16) show the co-localization of DISC1 (green) and NESTIN (red). In (b20) and (b24), the merged images represent the co-localization of TRIAD1 and DISC1 in the nuclei of NSCs

Inflammatory activation of astrocytes is an important pathological change after brain injury. Therefore, we set up a co-culture model with NSCs isolated from embryonic day 16 (E16) rat cortex and astrocytes stimulated by lipopolysaccharide to simulate the process of NSCs’ mobilization after TBI. Then we extracted a centimeter of astrocytes and co-cultured with neural stem cells. Through immunofluorescence, we discovered TRIAD1, DISC1, and NESTIN can co-localize with each other in NSCs in primary normal conditions and co-cultured with LPS-astrocytes (Fig. 4).

### The relationship between TRIAD1 and DISC1 and their specific domain interactions

**Fig. 5:**
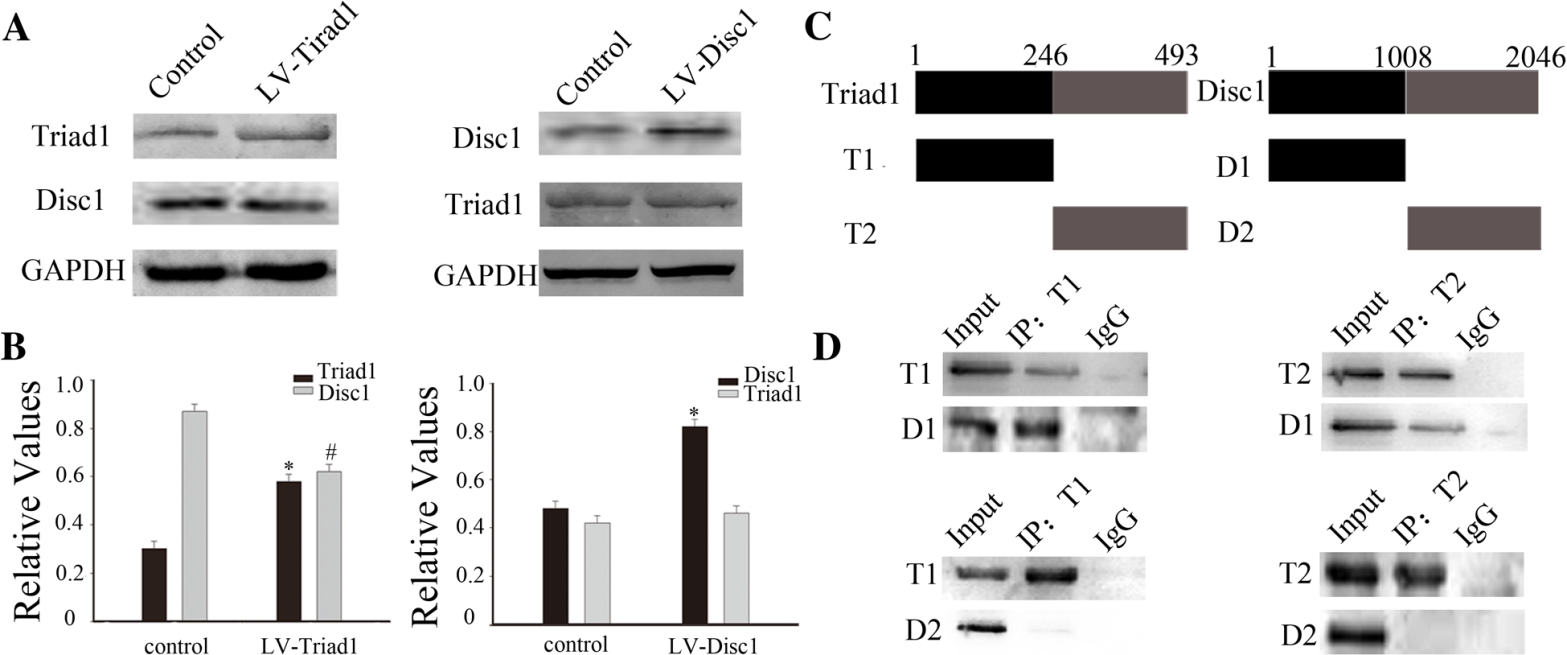
The relationship between TRIAD1 and DISC1 and their specific domain interactions. **a** TRIAD1 or DISC1 was transfected alone into 293T cells, and then examined another through Western blot. **b** is the histogram (relative optical density) quantifying the intensity of **a**. **c** Truncated plasmid structure diagram of two molecules. **d** Immunoprecipitation showed that the specific domain interaction of TRIAD1 and DISC1

To clarify which protein plays the more dominant role, we transfected TRIAD1 or DISC1 alone into 293T cells and examined their effect on each other. We observed that the expression of DISC1 decreased after overexpression of TRIAD1, whereas the expression of TRIAD1 changed very little after overexpression of DISC1 (Fig. 5a, b).

To further determine the details of the interaction between TRIAD1 and DISC1, we constructed two plasmids containing different truncated versions (S1 and S2) of each protein (Fig. 5c), and co-transfected them into 293T cells for detection by co-IP. DISC1-S1 immunoprecipitated with TRIAD1-S1 and TRIAD1-S2, while DISC1-S2 showed no obvious positive results. This indicates that DISC1 and TRIAD1 can interact via specific protein domains (Fig. 5d).

### Effect of bimolecular interactions on the proliferation and differentiation of neural stem cells

**Fig. 6:**
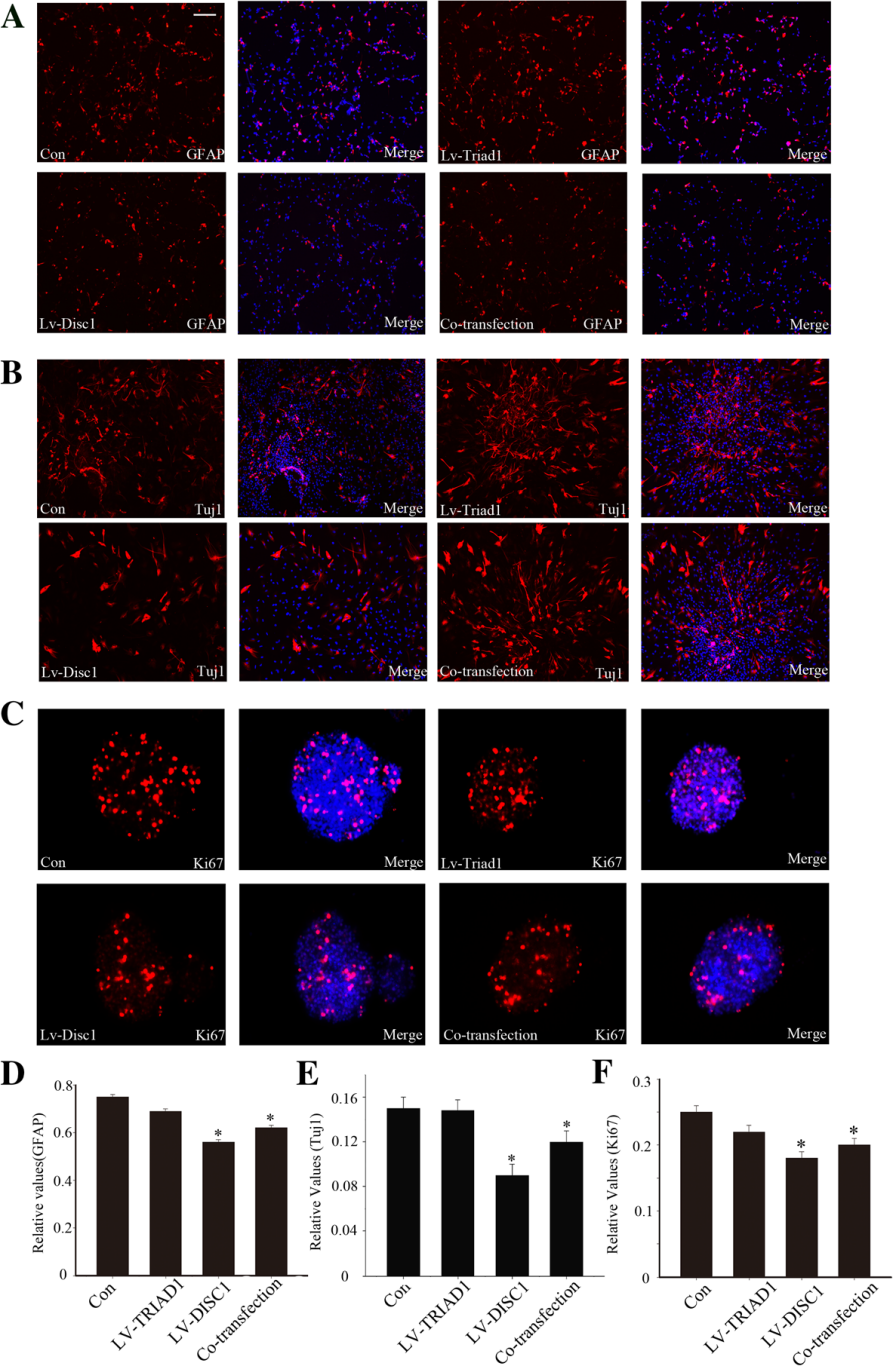
Effect of bimolecular interactions on the proliferation and differentiation of NSCs. **a**–**b** Neurospheres were treated by the different methods mentioned above and then detect the amount of GFAP-positive cells and Tuj1-positive cells. Scale bars: 50 μm. **c** The amount of Ki67-positive cells was observed with a confocal microscope. Scale bars: 50 μm. **d**–**f** Quantitative analysis of GFAP-positive cells, Tuj1-positive cells and Ki67-positive cells compared with DAPI. * indicates a significant difference at *P* < 0.05 compared with the control group. Error bars represent the SEM

Inflammatory activation of astrocytes is an important pathological change after brain injury. Therefore, we co-cultured the embryonic NSCs with stimulated astrocytes. We then transfected the NSCs with lentiviral vectors containing either DISC1 (LV-DISC1) or TRIAD1 (LV-TRIAD1) or both. Four days after induction of differentiation, we identified differentiation phenotypes under a confocal microscope by immunofluorescence labeling with GFAP (marker for astrocytes) and Tuj1 (marker for neurons). We found that the number of GFAP-positive cells and Tuj1-positive cells was reduced in cells transfected with LV-DISC1, while the LV-TRIAD1 group was similar to control. Interestingly, the number of GFAP-positive cells in NSCs transfected with both LV-TRIAD1 and LV-DISC1 was more than that in the LV-DISC1 group and less than that in the control group and LV-TRIAD1 group (Fig. 6a, b). Moreover, the pattern of proliferation, marked by Ki67-positive NSCs, was similar to the differentiation of NSCs (Fig. 6c). These results show that DISC1 inhibits the proliferation and differentiation of NSCs. TRIAD1 has no obvious effects directly on differentiation or proliferation of NSCs but seemed to dampen the effect of DISC1.

## Discussion

With the continued progress of society, events like car accidents and falls, which can cause brain injury, are increasing every year. The increased severity of these injuries amplifies the probability of neuronal damage, specifically neuronal apoptosis and necrosis. Several studies have shown that that neuronal necrosis is irreversible, and neurons cannot be regenerated [26–28]. Accordingly, mobilization and multi-directional differentiation of NSCs after brain injury is a point of focus for research in the TBI field.

In this study, we investigated the expression of two molecules, TRIAD1 and DISC1, and the changes in cytology after brain injury in a rat model of TBI. Western blots and immunohistochemistry showed that the expression of TRIAD1 was increased, and the expression of DISC1 was decreased in the hippocampus at the same time point after injury. Immunofluorescence in tissue sections revealed that these two proteins co-localize in the dentate gyrus. Furthermore, immunofluorescence in culture showed that TRIAD1 and DISC1 also co-localize in NSCs. Finally, co-IP results revealed an interaction between TRIAD1 and DISC1 after brain injury in rats. In corroboration with the above findings, we were also able to detect both proteins in primary NSCs isolated from the hippocampi of 1-day-old rats. Overexpression of TRIAD1 or DISC1 via lentiviral transfection demonstrated their different roles in NSC differentiation and proliferation, while co-IP of truncated versions of each protein narrowed down the specific domains responsible for this bimolecular binding.

According to recent studies, DISC1 is considered to be the primary susceptibility gene for schizophrenia [29]. It is widely present in the brain of various organisms, especially during embryonic nerve development [30, 31]. As an important player in complex signaling cascades, DISC1 participates in various physiological processes of the cell, especially those involving brain development. DISC1 can also influence the proliferation and differentiation of NSCs by interacting with molecules such as Dixdc1, Fez1, Sox2, and Sox10. It has been shown to play a role in the canonical Wnt signaling pathway and regulate the proliferation of neural precursor cells [32, 33]. Along with unphosphorylated Dixdc1, DISC1 participates in the regulation of the Wnt-GSK3β/β-catenin signaling pathway to promote proliferation of neural precursor cells [34, 35]. Moreover, in the presence of DISC1, phosphorylated Disxdc1 can bind another protein, NDEL1, to form a protein complex of DISC1/Dixdc1/NDEL1 that regulates the migration of cerebral cortical neurons [12]. It was reported that the presence of DISC1 can inhibit the differentiation of progenitor cells [36, 37]. In the hippocampi of rats, a knockout of the DISC1 gene causes neural precursor cells to have much larger somas and longer axons than in untreated rats [38]. There are many reports of the role of DISC1 in oligodendrocytes, neural crest cells, and other cell types [37, 39]; however, no clear report exists on the role of DISC1 in the mobilization and differentiation of NSCs after brain injury. How does DISC1 regulate the migration of NSCs and their differentiation into neurons and glia? Which specific domains of DISC1 are critical to downstream biological functions? We sought to answer these questions in our study and found that TRIAD1 can interact with DISC1 to regulate the proliferation and differentiation of NSCs. Despite their influence on critical cellular processes like proliferation and differentiation, the effect of TRIAD1 and DISC1 on other biological functions of NSCs has yet to be examined.

Ubiquitination is a widespread process in most cell types in the body [40]. Studies have shown that the expression levels of many different molecules change after brain injury and affect nerve cell function [18, 41]. Likewise, we found in this study that expression levels of TRIAD1 and DISC1 change after TBI and affect the cellular functions of proliferation and differentiation. To offset the proteins that increase expression, like TRIAD1, the cell ubiquitinates and targets them for degradation. As in TBI, ubiquitination also plays a big role in diseases, such as cancer and cardiovascular disease [42, 43], and has thus become a popular target for the development of new drugs [44]. Our study did not examine the ubiquitination and degradation mechanisms of TRIAD1 after TBI; our research group has previously examined only the change in total ubiquitination levels after brain injury. The specific ubiquitination sites, as well as the other E1 and E2 ubiquitin enzymes involved in the degradation of TRIAD1, remain unclear. As an E3 ubiquitin ligase, TRIAD1 is an important part of the cell’s UPS, and aberrant increases in its expression can have significant downstream effects. Marteijn and colleagues found that two ubiquitin-binding enzymes, UbcH7 and Ubc13, can respectively bind two zinc-finger domains in the TRIAD1 protein. Their experimental results indicate that TRIAD1 can be ubiquitinated through the binding of UbcH7 and Ubc13, thereby influencing bone marrow cell formation [22]. Therefore, we hypothesized that the two zinc finger domains of TRIAD1 would also be key domains of interaction with DISC1. Although we observed no significant change in the subcellular localization of TRIAD1 or DISC1 after injury, we found that they can interact with each other. This interaction could in turn lead to sub-localization of their unknown substrate(s) and corresponding biological changes. In summary, we studied the expression of TRIAD1 and DISC1 after brain injury, their localization and impact on NSC differentiation and proliferation, and the specific domains involved in their interaction. We discovered that TRIAD1 can interact with DISC1 after TBI. Their interaction is of great significance to the repair of central nervous system injury via the regulation of proliferation and differentiation of NSCs. Our data presents a potential therapeutic target for the treatment of TBI by elucidating the molecular mechanisms of NSC mobilization after brain injury. In future studies, we will examine their ubiquitination mechanisms, as well as clarify their specific mechanisms of function in NSC proliferation and differentiation after neural injury.

## Conclusions

In this study, we found that the expression of TRIAD1 increased progressively, reached a peak at 3 to 5 days, and then decreased gradually. While the expression level of DISC1 was correlated with TRIAD1, its expression level at 3 days was significantly lower than at other time points. Immunofluorescence on frozen brain sections showed that TRIAD1 and DISC1 are co-localized in neural stem cells. Immunoprecipitation data suggested that TRIAD1 may interact with DISC1. Finally, we found that overexpressing TRIAD1 and DISC1 significantly affected the proliferation and differentiation of those NSCs. Taken together, these data show that the expression of TRIAD1 and DISC1 change after traumatic brain injury and that their interaction may affect the proliferation and differentiation of neural stem cells.
